# A systematic review of the direct and indirect COVID-19’s impact on food security and its dimensions: pre-and post-comparative analysis

**DOI:** 10.1186/s12889-023-17104-6

**Published:** 2023-11-20

**Authors:** Daniel Teshome Gebeyehu, Leah East, Stuart Wark, Md Shahidul Islam

**Affiliations:** 1 School of Health, University of New England, Armidale, Australia; 2 School of Veterinary Medicine, Wollo University, Dessie, Ethiopia; 3 School of Nursing and Midwifery, University of Southern Queensland, Toowoomba, Australia; 4 School of Rural Medicine, University of New England, Armidale, Australia

**Keywords:** COVID-19 impact, Food insecurity, Food security dimension, Systematic review

## Abstract

**Background:**

Since its emergence, the COVID-19 pandemic has compromised the food security both directly by impacting food supply chain and indirectly by overwhelming the individual health and/or personal financial situation. The overarching aim of the current study is to assess aspects of the food security crisis that have arisen due to COVID-19 and to identify which, if any, food security dimensions were specifically compromised.

**Methods:**

Primary research articles were initially identified through four online databases (Scopus, PubMed, Google Scholar, and Web of Science), with the references of each paper then also reviewed for additional article. The food security status of individuals and the wider community, both before and after the emergence of COVID-19, were examined.

**Results:**

Of the 2,057 studies initially identified, a total of ten were included in the final review. The included studies confirmed that COVID-19 had substantially impacted food security, with individuals, households and the wider community experiencing food insecurity. Nine of the included studies aruged that the food accessibility dimension was the most compromised.

**Conclusion:**

To address the identified direct and indirect food security issues associated with COVID-19, it is proposed that a combination of prevention practices and proactive food security activities is required. Integrating food security interventions, supporting and facilitating food security resilience, and conducting further studies on the food security of COVID-19 are also recommended.

**Supplementary Information:**

The online version contains supplementary material available at 10.1186/s12889-023-17104-6.

## Introduction

Food security is defined as having healthy, nutritious, and physically and economically accessible food available to people in a defined geographic location and which satisfies the individuals’ dietary needs [1]. COVID-19, a communicable disease caused by the viral pathogen SARS-COV-2, continues to have multi-dimensional impacts all over the world [2–4]. Since its nominal emergence (31 December 2019), the COVID-19 pandemic has affected the food security of people across the world [5–9]. Specifically, the pandemic has impacting the food security of individuals and communities both directly and indirectly by overwhelming the agricultural productivity, exacerbating the energy poverty [10], and diminishing personal health and financial stability [11–13]. Prior to COVID-19, approximately 820 million individuals faced hunger each day globally, with over two billion individuals experiencing micronutrient deficiencies. These dietary issues in turn lead to increased risk of mortality and morbidities [14, 15]. COVID-19’s emergence resulted in disruptions to the production, processing, transporting, and marketing of food [15], which has caused flow-on impacts to food security. For example, fresh food products, including meat, fish, fruits, and vegetables, all recorded extensive price increases due to obstructions in transport channels [16]. A joint statement released in 2020 by the World Health Organization, (WHO) amongst others [14], reported that 3.3 billion global workers were identified as being at risk of losing their livelihood due to COVID-19. COVID-19 had exacerbated the already existing malnutrition in children. Further, 149 million children were estimated to have growth abnormalities, such as stunted (reduced in their growth performance), globally due to COVID-19 and other confounding factors, including natural disasters like drought, desert locust emergence, war and displacement [17]. The food security issues that arose due to COVID-19 were felt globally [18, 19], however, the level of crisis was more significant in lower-income countries [15] than in middle- and high-income countries.

According to the Food and Agricultural Organization (FAO) of the United Nations (UN) [1], there are four food security dimensions: availability; accessibility; usability; and sustainability/stability. Food availability is defined as the presence of adequate amount of food that satisfies the demand of the general population within a specified territory [20], while accessibility is the retrievability of food without excessive physical and/or economic barriers [21]. Usability of food refers to the nutritional quality of food and focuses on whether the accessible and available food is balanced, healthy, and nutritious [21]. This available and accessible food should then be able to satisfy the dietary and nutritional demands of the individual, households, community and/or the general population [20–22]. This food should be sustainable/stable by being available, accessible, and useful for a prolonged time [15, 20, 21]. All the four dimensions of food security are interdependent to each other and the effects of a certain factor that impacts one dimension can directly or indirectly overwhelm another [1]. As such, it is not appropriate to talk about food security using only one food security dimension. For instance, it is meaningless to discuss food accessibility, stability, and usability if food is not available and it is not rational to talk about food usability if food is not accessible.

A recent article has identified the burden of COVID-19 pandemic on all the four dimensions [23], while another examined some of the food security dimensions [24]. Interestingly, findings from Iran showed that the emergence of COVID-19 had short term positive impact on food security of households and individuals [25]. Studies on the impact of COVID-19 were conducted in different geographic locations of the world, but the findings are currently inconsistent and sometimes contradictory. As a result, a compiled summary of all the available information, and which is informative for policy makers, food input suppliers, aid agencies and governmental and non-governmental bodies, is considered an appropriate research goal. In addition, identifying whether (and which) different food security dimensions have been impacted to greater or lesser extents during COVID-19 may assist local, regional and national government bodies in mitigating any emerging food insecurity issues.

To achieve these outcomes, a systematic review was conducted of all studies regarding COVID-19’s impact on food security. This paper examines the extent to which food security was directly and indirectly compromised by COVID-19, and how food security actors recovered from the COVID-19 pandemic induced food insecurity trauma. The application of findings of this systematic review are not necessarily restricted explicitly to COVID-19 as they may also have potential significance in early preparedness of the inevitable future pandemics. The purpose of this article was to summarise the food security impact of COVID-19 and pointing out the critically impacted dimension/s of food security. The specific questions that guided this systematic review were:

1. How much was food security directly and indirectly affected by COVID-19?
2. Which, if any, dimensions of food security were significantly compromised by the COVID-19 pandemic?

### Methodology

This systematic review was undertaken in line with the PRISMA 2020 guidelines [26] and checklist [additional file 1]. In addition, the Joanna Briggs Institute (JBI) systematic review checklist was used as a second point of reference to guide the process. Prior to the systematic review commencing, a protocol was prepared and registered in ROSPERO database with registration number CRD42022325475 and it was published online [27]. As indicated in the following sub-headings, each aspect of the PRISMA checklist was addressed sequentially.

### Eligibility criteria

Primary research articles conducted on COVID-19’s impact on food security were the focus of the systematic review. However, so as to allow for comparative analysis of the burden of COVID-19, potential articles had to specifically focus on at least one of the four defined dimensions (food availability, accessibility, usability and stability) both prior to and/or following the emergence of COVID-19. No restrictions were placed on study design, with any qualitative, quantitative, and mixed method studies considered. Other types of literature (grey literature, review articles, protocols, reports, letters to editors and unpublished studies) were excluded from review. The reasons for excluding information sources other than primary research articles were to ensure the goal of identifying information from the primary source, and which were supported with data and followed expected research standards and procedures.

It is expected that primary research articles published in different languages on the topic of interest might be available in online information sources (databases), but only studies written in English were included. The timeframe for publication was from the approximate time of emergence of COVID-19 (31 December 2019) through to the end of this systematic review’s literature search (15 May 2022) were included.

### Information sources, search strategy, and selection process 

Four databases (Web of Science; Scopus; PubMed; and Google Scholar) were used as the literature sources. The data from these four databases was identified from the nominal date for COVID-19 pandemic emergence (31/12/2019) through to 15/05/2022. All reference lists from the studies identified through the initial database searches were then explicitly assessed to avoid missing any relevant data source.

A combination of search terms was used. The same terms were used for three databases (PubMed, Scopus, and Web of Science), and search strings were applied according to the relevant database. As explained in the protocol [27], the search terms used for Web of Science, Scopus, and PubMed were *“Impact” OR “Effect” OR “Burden” AND “COVID 19”) OR “COVID-19 pandemic” OR “SARS COV 2” OR “Coronavirus disease 2019” OR “Coronavirus diseases 19” OR “Severe acute respiratory syndrome coronavirus-2”) AND “Food Security” OR “Food Insecurity” OR “Food Security dimension”*. The publication date (31/12/2019—15/05/2022), the publication language (English), and the article type (primary research articles) were used as a filtering mechanism during the searching process. For identifying literature from Google Scholar, the short title of this systematic review (*the COVID-19’s impact on food security, availability, accessibility, usability and stability*) was used, and any potentially relevant articles that were not identified from other databases were included. Details of the search strategy for each database is described as follows.

- Scopus: ( ALL ( impact) OR TITLE-ABS-KEY ( effect) OR TITLE-ABS-KEY ( burden) AND ALL ( covid 19) OR ALL ( covid-19 AND pandemic) OR TITLE-ABS-KEY ( sars AND cov 2) OR TITLE-ABS-KEY ( coronavirus AND disease 2019) OR TITLE-ABS-KEY ( coronavirus AND diseases 19) OR ABS ( severe AND acute AND respiratory AND syndrome AND coronavirus-2) AND ALL ( food AND security) OR ALL ( food AND insecurity) OR TITLE-ABS-KEY ( food AND security AND dimension)) AND ( LIMIT-TO ( DOCTYPE, "ar")) AND ( LIMIT-TO ( PUBSTAGE, "final")) AND ( LIMIT-TO ( LANGUAGE, "English")).
- PubMed: (((((((((((Impact[Title/Abstract]) OR (Effect[Title])) OR (Burden[Title])) AND (COVID 19[Title/Abstract])) OR (COVID-19 Pandemic[Title/Abstract])) OR (SARS COV 2[Title/Abstract])) OR (Coronavirus disease 2019[Title/Abstract])) OR (Coronavirus diseases 19[Title/Abstract])) OR (Severe acute respiratory syndrome coronavirus-2[Title/Abstract])) AND (Food Security[Title/Abstract])) OR (Food Insecurity[Title/Abstract])) OR (Food Security dimension[Title/Abstract]).
- Web of Science: Impact (All Fields) OR Effect (Abstract) OR Burden (Abstract) AND COVID 19 (All Fields) OR COVID-19 pandemic (All Fields) OR SARS COV 2 (Title) OR Coronavirus disease 2019 (All Fields) OR Coronavirus disease 19 (All Fields) OR Severe acute respiratory syndrome coronavirus-2 (Abstract) AND Food Security (All Fields) OR Food Insecurity (All Fields) OR Food Security dimension (Abstract) and 2020 or 2021 or 2022 (Publication Years) and Article (Document Types) and English (Languages).
- Google scholar: COVID-19, Food Security, Impact, OR COVID-19, OR Pandemic, OR Food OR security, OR availability, OR accessibility, OR usability, OR stability.

The first author conducted the primary screening, and the same process was repeated by the other team members to ensure the validity and reliability of selection processes. Identification of potentially eligible studies was conducted initially through title and abstract screening, prior to a full text review occurring. A pre-determined process for resolving any disagreements was established, however ultimately there were no disagreements in the selection process among the review authors.

### Data items and collection process 

Studies selected from the four databases were imported to EndNote X9. Literature searches were undertaken on 01 May 2022 for the first time and repeated 2 weeks later for the second time. The identified articles were de-duplicated using EndNote X9’s unique identifier function. Any redundant studies missed by the EndNote unique identifier were manually de-duplicated. The studies were considered regardless of the studies’ statistical analysis, geographic coverage, and study participants (individual, household, community or country). All COVID-19 related factors that exacerbated the food insecurity of individuals/households, such as COVID-19 prevention restrictions, agricultural production interruptions, job losses, import–export bans, and mortality of productive workforces, were synthesised into the final results.

### Study risk of bias assessment 

The Joanna Briggs Institute (JBI) critical appraisal checklist was used for assessing the risk of biases. The eight items of the JBI checklist are designed for assessing the risk of biases in reviewing primary studies with *Yes, No, Unclear or Not Applicable* answers. The items of the checklist are: 1) the inclusion and exclusion criteria of samples; 2) study subjects and settings; 3) exposure measurement validity and reliability; 4) objective and standard criteria of condition measurement; 5) identification of confounding factors; 6) strategies to deal with confounding factors; 7) validity and reliability of outcome measures; and 8) the use of appropriate statistical analysis in included studies.

Based on the JBI critical appraisal checklist, each included study was independently appraised by a second author and no disagreement were raised among team members.

### Effect measures and synthesis methods

COVID-19-related food production or supply chain restrictions was considered as impacts on food availability. Food security reductions due to physical or economic constraints during COVID-19 were classified as food inaccessibility. The food quality and diversity reduction during the pandemic was interpreted as the impact of COVID-19 on food usability. Short-term food items availability that cannot be sustained due to the direct (effect on the food supply chain) and indirect (health and economic burdens) impacts were deemed to be food stability issues. Food insecurity increases during COVID-19 as compared with pre-pandemic food insecurity were expressed in the form of percentages. Overall, one or more food security dimension/s that was/were repeatedly reported by the majority of the included studies was identified as significantly altered dimension by COVID-19 pandemic.

Synthesis of this systematic review was focused on investigating COVID-19’s impact on food security and/or its four dimensions (availability, accessibility, usability and stability). Any included studies that failed to consider relevant confounding food insecurity drivers were critically appraised in the discussion and risk of bias assessment sections of this systematic review.

### Reporting bias and certainty assessments

The Agency for Healthcare Research and Quality (AHRQ) tool was used for assessing reporting biases in the studies. As recommended by Berkman et al. [28], the authors independently assessed the risk of bias in the papers using the AHRQ checklists. Based on the checklists, the studies with a high probability of reporting bias were categorised as “suspected” and the studies with a low probability of bias were noted as “undetected.” The certainties of evidence were assessed using the GRADE tool. The alignment of findings, the consistency of evidence, the level of suspected publication biases, the limitations of reviewed studies, and the availability of target outcomes in the reviewed studies were considered as factors for certainty.

## Result

### Study selection

**Fig. 1:**
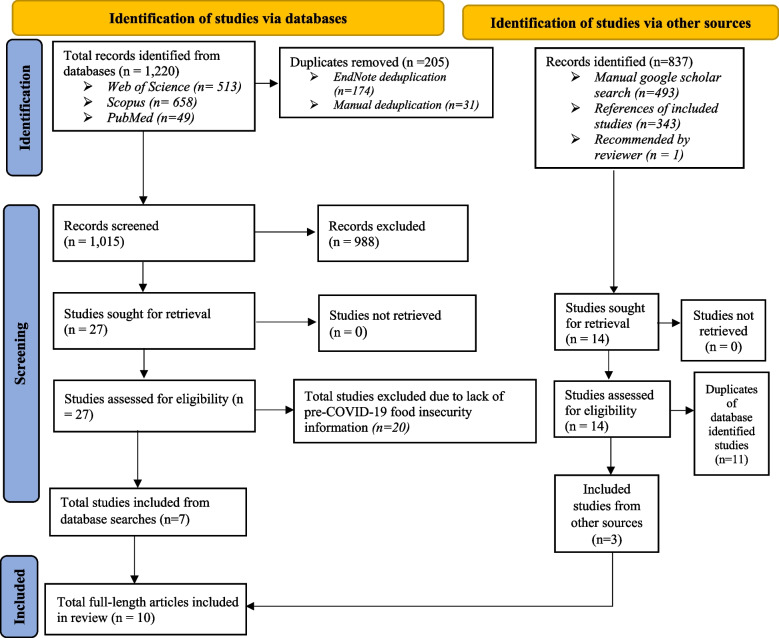
Study selection process and sources using the PRISMA 2020 model

Of the initial sample of 2,057 studies identified through the first search, 1,220 were found through database searching and the remaining 837 were from other sources, such as the aforementioned review of reference lists (Fig. 1). Of the 1220 papers found through database searches, 27 were identified as eligible for review, but 20 were removed due to the lack of pre-COVID-19 food security information. Fourteen potentially eligible studies were identified from other sources; 11 of these were removed because they were duplicates of studies already identified. Overall, this resulted in seven studies from the database search and three studies from other sources being included in the final sample of ten papers (Fig. 1). The study by Pradeilles et al. [29] in Peru was initially considered eligible to be included for review, but was removed due to a lack of pre-COVID-19 data for the food insecurity experience scale (FIES) indicator.

### Characteristics of the reviewed studies

Two papers were published in 2020, seven in 2021, and one in 2022 (Table 1). Three studies [18, 30, 31] were conducted in the United States of America and the remaining studies included countries such as Indonesia, Bangladesh, Uganda, Kenya, Chile, Mexico, Lebanon, and Iran (Table 1). Only one study [16] covered the food insecurity prevalence of two countries (Kenya & Uganda), with the others covering either a single county or a province [25, 32] in a single country, or populations [30, 31] in the same country.

**Table 1:** General characteristics of the reviewed studies

Studies	Country	Study population	Sample size	Study duration	Study design	Comparison
Ahmed et al. [30]	USA	College students	1,989	Fall semester 2019 to end of fall semester 2020	Cross-sectional	The food security status before pandemic with the food security status after a pandemic
Faridi & Furqan [32]	Indonesia	Households	218	May—June 2020	Cross-sectional	Food insecurity levels before COVID-19 with during COVID-19
Gaitán-Rossi et al. [33]	Mexico	Households	3,357	April—June 2020	Cross-sectional	National survey 2018 result with food insecurity result during COVID-19
Giacoman et al. [34]	Chile	Households	70,677 pre COVID-19 & 4,425 during COVID-19	24 June—7 August 2020	Cross-sectional	2017/18 food insecurity level with food insecurity level during COVID-19
Hamadani et al. [35]	Bangladesh	Mothers	3, 016 Children’s mother	19 May—18 June 2020	Interrupted time series	The status of food insecurity in a median of 1 and 2 years pre-COVID-19 with the status of food insecurity post-COVID-19
Kansiime et al. [16]	Kenya & Uganda	Individual respondents	442	18–27 April 2020	Cross-sectional	Food insecurity level before pandemic with during pandemic
Kharroubi et al. [36]	Lebanon	Individual adults	3, 000	Retrospective data from 2015—2017	Predictive model	Food insecurity estimates before COVID-19 with food insecurity estimates during COVID-19
Mialki et al. [31]	USA	College students	3, 206	April—May 2020	Cross-sectional	Food security status before and after the onset of the COVID-19 pandemic
Niles et al. [18]	USA	Adult individuals	27,168	March to May 2020	Cross-sectional	Food insecurity before pandemic with during pandemic
Pakravan-Charvadeh et al. [25]	Iran	Households	299	March to February 2020	Cross-sectional	Dietary diversity and food security status of households before and after the emergence of COVID-19

The summarised objective of all reviewed studies was to determine COVID-19’s burden on food security and associated dimension/s using the pre and post-COVID-19 food security analysis (Table 1). Four studies [25, 32–34] assessed the food security levels of households, three [16, 18, 36] identified the effect of the pandemic on the food security of individuals, while two studies [30, 31] investigated food insecurity levels in college students before and at the time of the pandemic in the United States of America. The remaining study [35] assessed food security of mothers before and after the pandemic in Bangladesh. The majority (8 out of 10) of the studies used a cross-sectional study design (Table 1) and the remaining studies used interrupted time series [35], and predictive model [36] designs. As indicated by Gaitán-Rossi et al. [33], food security of households with children was reduced. Contrary to the majority (9 out of 10) of the studies, the research conducted in Iran’s Tehran province [25] reported that the emergence of COVID-19 resulted in improved food security among households.

### Risk of bias in studies

Based on the JBI critical appraisal checklist for cross-sectional studies, six studies were assessed as having a low risk of bias in all 8 items of the checklist while 4 studies [25, 32, 35, 36] were found to have unclear risk of bias in four items (Q1, Q3, Q4, and Q6) of the checklist (Table 2). Eight of the included studies used cross-sectional study design and the remaining two studies [35 and 36] had used predictive model and interrupted time series study designs. The JBI critical appraisal checklist for cross-sectional studies was designed for analytical cross-sectional studies and it was challenging to find a specific and appropriate risk of bias assessment tool for the two studies that used a predictive model [36] and interrupted time series [35]. Since the main difference of these study designs from cross-sectional methodologies is time-frame and ways of measurement, the JBI critical appraisal checklist for cross-sectional studies was considered still potentially suitable for predictive model and interrupted time series designs, and was therefore used.

**Table 2:** Included studies’ risk of bias assessment results

Studies	Assessment questions/checklist								Over all appraisal
	**Q1**. Were the criteria for inclusion in the sample clearly defined?	**Q2**. Were the study subjects and the setting described in detail?	**Q3**. Was the exposure measured in a valid and reliable way?	**Q4**. Were objective, standard criteria used for measurement of the condition?	**Q5**. Were confounding factors identified?	**Q6**. Were strategies to deal with confounding factors stated?	**Q7**. Were the outcomes measured in a valid and reliable way?	**Q8**. Was appropriate statistical analysis used?	
Ahmed et al. [30]	Yes	Yes	Yes	Yes	Yes	Yes	Yes	Yes	IN
Faridi & Furqan [32]	Yes	Yes	Yes	Yes	Yes	Unclear	Yes	Yes	IN
Gaitán-Rossi et al. [33]	Yes	Yes	Yes	Yes	Yes	Yes	Yes	Yes	IN
Giacoman et al. [34]	Yes	Yes	Yes	Yes	Yes	Yes	Yes	Yes	IN
Hamadani et al. [35]	Yes	Yes	Unclear	Yes	Yes	Unclear	Yes	Yes	IN
Kansiime et al. [16]	Yes	Yes	Yes	Yes	Yes	Yes	Yes	Yes	IN
Kharroubi et al. [36]	Yes	Yes	Unclear	Unclear	Yes	Yes	Yes	Yes	IN
Mialki et al. [31]	Yes	Yes	Yes	Yes	Yes	Yes	Yes	Yes	IN
Niles et al. [18]	Yes	Yes	Yes	Yes	Yes	Yes	Yes	Yes	IN
Pakravan-Charvadeh et al. [25]	Unclear	Yes	Yes	Yes	Yes	Yes	Yes	Yes	IN

^Answer alternatives for each item are^^: Y^^es, No, Unclear, Note Applicable (N/A)^

^Over all appraisal alternatives are^^: i^^nclude (IN), exclude (EX), seek further information (SFI)^

^Q stands for “question”^

### The compromised food security dimension and its associated factors 

A majority (9 out of 10) of the studies used validated food security indicators, while one [36] of the studies estimated the food insecurity prevalence using a predictive model from previous food insecurity trends. The most compromised food security dimension identified across nine studies was the accessibility of food, with all the studies reporting that economic crisis due to COVID-19 was the main cause of food inaccessibility (Table 3). Two studies [16, 32] confirmed that the usability (quality) of food was reduced during COVID-19 and that food diversity during COVID-19 was less than the food diversity before the emergence of the pandemic. The study from Iran [25] reported that the food diversity (usability/quality) of households had actually increased following COVID-19 and the food security was improved. This was in contrast to the study conducted in Indonesia [32] which indicated that food access, availability, and usability were all compromised by COVID-19.

**Table 3:** Identifying the compromised food security dimension (dimensions) due to COVID-19

Studies	Food insecurity measure/indicator	Compromised dimension	Identified factors for the compromised dimension
Ahmed et al. [30]	AFSSM	Food access	➢Lack of access to the food support programs during COVID-19 ➢Low economic capacity ➢No food access options during the pandemic
Faridi & Furqan [32]	ELCSA	Food access, availability & usability	➢Economic crises due to COVID-19 ➢Closure of restaurants and food shops ➢Interrupted rural food transport
Gaitán-Rossi et al. [33]	ELCSA	Food access & availability	➢Health and social anxiety ➢Economic crises due to job lost ➢Lack of physical access
Giacoman et al. [34]	FIES	Food access	➢Economic crises due to job lost ➢Declined income
Hamadani et al. [35]	HFIAS	Food access	➢Reduction in paid work and income ➢Socio-economic crises
Kansiime et al. [16]	FIES	Food access & usability	➢Income shock during COVID-19 ➢Disruption of rural–urban food market chain
Kharroubi et al. [36]	Food security trend analysis	Food access	➢Economic crisis due to COVID-19
Mialki et al. [31]	AFSSM	Food access	➢Changes in housing and employment status due to the pandemic
Niles et al. [18]	USDA ERS	Food access	➢The high number of family members ➢Job disruption and income reduction
Pakravan-Charvadeh et al. [25]	HFIAS and HDDS	No compromised dimension	➢Free food supplement to vulnerable group, extending e-marketing, delivering nutrition advice, and donations to the victims increased dietary diversity and improve food security the during a pandemic

*AFSSM* = Adult Food Security Survey Module, ELCSA = Latin American and Caribbean Food Security Scale, *FIES* = Food Insecurity Experience Scale, *HFIAS* = Household Food Insecurity Access Scale, *USDA ERS* = United States Department of Agriculture Economic Research Service, *HDDS* = Household Dietary Diversity Score

Two studies [30, 31] examined food security using prior and following the pandemic emergence food security levels of individuals. Both papers identified the negative impact of COVID-19 on food security, with food security status reduced during the pandemic as compared with the pre-pandemic food security status (Table 4). The remaining eight studies explored COVID-19 related food security using pre- and post-COVID-19 emergence food insecurity prevalence.

**Table 4:** Before and after COVID-19 emergence food insecurity/security status variations

Country	Pre-COVID-19 food insecurity	During COVID-19 food insecurity	Indicator
Ahmed et al. [30]	26.93%	23.59%	AFSSM (Low food security)
Faridi & Furqan [32]	4.819 ± 5.3	19.775 ± 7.3	ELCSA (Average food insecurity ± standard deviation)
Gaitán-Rossi et al. [33]	31% (2018)	42% (May 2020)	ELCSA (Mild Food insecurity)
Giacoman et al. [34]	30 (2017–2018)	49 (2020)	FIES (Average food insecurity status)
Hamadani et al. [35]	5.6% (2017–2019)	36.5% (May–June 2020)	HFIAS (Moderate food insecurity)
Kansiime et al. [16]	50% Kenya & 43% Uganda	88% Kenya and 87% Uganda (April 2020)	FIES (% of food insecure households)
Kharroubi et al. [36]	27%	36%—39%	Food insecurity trend analysis (Estimated food insecurity)
Mialki et al. [31]	56.1%	40.4%	AFSSM (Food security status)
Niles et al. [18]	21.8%	30.2%	USDA ERS (Cumulative food insecurity status)
Pakravan-Charvadeh et al. [25]	35% food secure	43% food secure	HDDS and HFIAS (% of food secure households) that indicated the positive impact of COVID-19 on food security

*AFSSM =* Adult Food Security Survey Module, *ELCSA* = Latin American and Caribbean Food Security Scale, *FIES* = Food Insecurity Experience Scale, *HFIAS* = Household Food Insecurity Access Scale, *USDA ERS* = United States Department of Agriculture Economic Research Service, *HDDS* = Household Dietary Diversity Score

### Risk of reporting bias in reviewed studies 

Each reviewed study was assessed using the AHRQ bias assessment criteria. Berkman et al*.* [28] noted that the reporting of bias assessment outcomes using this tool shall be either “suspected” or “undetected”. If the reviewer cannot identify any reporting bias from studies, it is recommended to say ‘undetected’ than to say ‘free from reporting biases’. Based on this tool (AHRQ), the reporting biases of the majority (9 out of 10) of the reviewed studies were assessed as “undetected” as indicated by the tick “√” symbol in Table 5. The study [36] that used a predictive model for estimating food insecurity was assessed as being “suspected” of bias, as the study was based on the food insecurity data 2–4 years before the occurrence of the pandemic and did not necessarily report on data indicative of the current situation immediately prior to and during the pandemic. Since there might be unidentified food insecurity variation during that longer time period, it was considered potentially problematic to compare the food insecurity during COVID-19 with the situation greater than 2 years prior to the pandemic’s emergence. As a result, the food insecurity report by Kharroubi et al. [36] was suspected of potential bias.

**Table 5:** Risk of reporting bias assessment result using the AHRQ tool

Studies	Types of reporting biases and judgments					
	Publication bias		Selective outcome reporting bias		Selective analysis reporting bias	
	Suspected	Undetected	Suspected	Undetected	Suspected	Undetected
Ahmed et al. [30]		√		√		√
Faridi & Furqan [32]		√		√		√
Gaitán-Rossi et al. [33]		√		√		√
Giacoman et al. [34]		√		√		√
Hamadani et al. [35]		√		√		√
Kansiime et al. [16]		√		√		√
Kharroubi et al. [36]	√		√		√	
Mialki et al. [31]		√		√		√
Niles et al. [18]		√		√		√
Pakravan-Charvadeh et al. [25]		√		√		√

### Certainty of evidence 

Based on the GRADE Pro handbook the certainty of results was assessed in three modalities (low, very low, moderate, and high)*.* Each study was judged based on the pre-set criteria (Table 6). The study that included pre and post-COVID-19 emergence food security data, identified impacted food security dimension/s, and used collected data instead of estimations was categorised as having a ‘high’ certainty of evidence. The evidence from the majority (9 out of 10) of the studies had high certainty of evidence. The remaining paper [36], which used retrospective data collected 2–4 years before the emergence of COVID-19, was categorised as having low certainty of evidence, particularly considering the influences and multifactored nature of food insecurity.

**Table 6:** Certainty of evidence assessment result based on GRADE handbook

Studies	Food security assessment criteria and judgments				Overall certainty
	Pre-COVID-19	During-COVID-19	Identify affected food security dimension	Using real data/not estimation	
Ahmed et al. [30]	High	High	High	High	High
Faridi & Furqan [32]	High	High	High	High	High
Gaitán-Rossi et al. [33]	High	High	High	High	High
Giacoman et al. [34]	High	High	High	High	High
Hamadani et al. [35]	High	High	High	High	High
Kansiime et al. [16]	High	High	High	High	High
Kharroubi et al. [36]	High	Very low	High	Very low	Low
Mialki et al. [31]	High	High	High	High	High
Niles et al. [18]	High	High	High	High	High
Pakravan-Charvadeh et al. [25]	High	High	High	High	High

## Discussion

Conceptually, food security is a complex and multifaceted issue [37], with four separate dimensions (food access, availability, usability, and stability) and is influenced by biological, social, and economic factors. As a result of its complexity, it is difficult for all dimensions of food security to be covered with any single food security indicator and a combination of tools should be used to examine the impact of COVID-19’s on food security [37, 38]. The current systematic review was designed to assess the COVID-19 related crisis on food security and associated food dimensions. This was undertaken using a pre-and post-comparative analysis by considering the date of COVID-19 emergence as the reference point. The main findings from this systematic review are presented below.

### Food insecurity due to COVID-19

Nine of the included studies reported that COVID-19 negatively affected food security and reported reduced food security status [30, 31] or increased food insecurity prevalence [16, 18, 32–36]. These articles noted that food security reduction in the form of four food security modalities (high food security, marginal food security, low food security, or very low food security) [9, 16, 35, 39]. In contrast to the other papers, the study conducted in Iran [25] reported that COVID-19 had a positive impact by increasing the food security and dietary diversity of households. The authors of this study [25] justified that the food security and dietary diversity improvement during COVID-19 was due to free food supplements to vulnerable groups, extending e-marketing, delivering nutrition advice, and governmental and non-governmental donations to the people, during the pandemic.

The largest food insecurity increase (44%) due to COVID-19 was observed in Uganda followed by Kenya [16] with a 38% food insecurity prevalence difference pre-and post-pandemic. This food insecurity prevalence was considered to be largely due to the rural–urban marketing channel disturbance of fresh food products like fish, meat, and vegetables [16]. Except the study conducted in Iran [25], other studies indicated that food security was reduced during COVID-19, with reported figures of 17% in Indonesia [32], 11% in Mexico [33], 19% in Chile [34], 31% in Bangladesh [35], 12% in Lebanon [36], and 8.4% in the USA [18]. In two studies, food security was reduced by 15.7% [34] and 3.34% [30] due to COVID-19 in the United States of America. The economic crisis due to COVID-19, increased unemployment [39], food value chain disturbances [40, 41], marketing channel interruptions [42, 43], and food service provider (hotels, restaurants, supermarkets, and retailers) closures [44] were mentioned as the main constraints of food security during COVID-19. These findings would indicate that proactive planning to facilitate more immediate intervention measures by governmental and non-governmental organizations toward food security resilience are needed for future pandemics [25].

### The compromised food security dimension 

The majority (9 out of 10) of the included studies confirmed that the most affected food security dimension was food access, which is consistent with other research [9, 45] that identified food access as being substantially impacted. It is acknowledged that the included studies didn’t use equivalent methods that equally measure all four of the food security dimensions, and this may have led to some diversity in data. Nonetheless, there was a consistent theme across the majority of papers. It was argued that food inaccessibility was primarily due to the poor economic capacity of consumers. This is not surprising; however, it is worth highlighting that physical food inaccessibility was also considered a result of lockdowns which led to a reduction in food access [30–33].

In addition to food accessibility, the availability of food [32, 33], and usability/quality of food [16, 32] were reported as being compromised due to COVID-19. The effect of COVID-19 on food usability was considered to be compromised due to a reduction in food diversity. Specifically, one paper [32] identified that three food security dimensions (accessibility, availability, and usability) were affected due to the restrictions and measures applied for the prevention and control of the COVID-19 pandemic. Once food accessibility or availability is compromised, food stability/sustainability is impacted by default [24, 46]. Lockdown measures to prevent the spread of COVID-19 were identified as the main factor for this reduction in food security [47], and this one aspect of food security impacted the entire food system chain [23].

### Limitation of the reviewed studies and the review process 

The studies did not necessarily take into account any potential confounding factors, such as illnesses other than COVID-19 [48], natural disasters during COVID-19 (desert locusts in Kenya, Uganda, and Lebanon, and floods in Bangladesh) [49], and climate change at the time of the pandemic [50]. Such factors will also have contributed to the reduction of food security and can therefore obscure the real effect of COVID-19. It is also worth recognising that experience-based food security measures are usually subject to recall biases [51]. Since the included studies used cross-sectional study designs, it is not possible to establish trends of food insecurity developments and to prioritise potential interventions.

Due to time constraints, only primary research articles were included, with brief reports, unpublished studies, reviews, and studies published in languages other than English excluded. This approach to study selection might have excluded relevant literature. This is important as the time delay from doing a study until results are presented in a journal may have meant that potentially relevant papers relating to a recent issue, such as COVID-19, had not yet had sufficient time to be published. Moreover, it is acknowledged that the initial selection system using title and abstract can also be prone to potentially missing relevant studies [52].

## Conclusion, recommendations and future implications 

The food security of households and individuals was severely compromised due to COVID-19 emergence. The health, agriculture and socio-economic crises following the COVID-19 pandemic indirectly impacted food security as compared with the pre-pandemic situations. The food accessibility dimension of food security was mainly impacted due to the pandemic. Contrary to the pandemic’s negative impact, integrated food aid interventions by private sectors, and governmental and non-governmental organizations improved food security during COVID-19. Based on this conclusion, the following recommendations were forwarded.

### Balancing COVID-19 prevention and food security crises

To implement either localised or widespread COVID-19 restrictions, such as transport bans, lockdowns, food market chain restrictions, and quarantine, the benefits should outweigh any problems that arise. This is particularly relevant for disadvantaged groups, particularly in low-income countries, where circumstances characterised by ‘hand to mouth’ meant entire communities cannot easily cope with any new food security crises, such as those arising due to COVID-19 prevention measures [53]. Therefore, any COVID-19 prevention restrictions should consider these social groups and balance the positives of reduced COVID-19 transmission arising from such restrictions, against the detrimental impact associated with food security [54].

Decisions that are unidirectionally focused (i.e. focusing on health crisis only) and implemented hastily without due consideration to all possibly factors, may have a catastrophic impact on food security. Unless these decisions are made in integrated manner (for instance in a ‘one health’ approach), emerging crises due to food insecurity might be as devasting as the personal health impact directly from COVID-19. Participating multi-disciplinary professionals, facilitating emergency food aid and SafetyNet programs, and prioritizing the pros and cons of COVID-19 prevention measures before implementation, should all be considered as potential strategies for balancing COVID-19’s prevention measures and arising food security crises.

### Integrated food security interventions

Food insecurity, arising either directly from the pandemic or indirectly due to restrictions applied for its prevention and control, should be mitigated by integrated interventions that are coordinated by governmental and non-governmental organisations, individuals, and institutions. Food security interventions can reduce mortality associated with food insecurity while balancing the need for COVID-19 prevention restrictions [55]. Although the data is limited, the food security crises during COVID-19 appear to be higher in low- and middle-income countries when compared to high-income countries. Therefore, along with the integrated COVID-19 prevention, collaborative food security intervention between countries is recommended as a proactive strategy to alleviate future problems associated with pandemics [56]. There was stepwise food insecurity increments in households with children [33]. Based on this food insecurity trend, it is recommended that food security actors (like food suppliers, aid and agricultural organizations) prioritise households with children. Households and individuals who were vulnerable for food security crises, including those with lower income, unemployed household member, female-leaded household, households with more children, and household members with lower educational level, are particularly highlighted for urgent intervention areas [34].

### Supporting food security resilience

This systematic review found that food security was severely affected by COVID-19. This compromised food security needs to be supported to recover, and communities that experience food insecurity should be proactively assisted to overcome both immediate and emerging crises. To achieve food security resilience, communities needed to be provided with the tools and resources to be more independent. Unless cooperative food insecurity resilience is applied, the vulnerable groups' morbidity and mortality due to food insecurity will be exacerbated [57].

### Future implications 

There is currently a lack of comparative data examining food security pre and post-COVID-19 emergence, and a more nuanced understanding of these complex issues can only be achieved with a greater focus on these areas. Studies regarding the effect of post-pandemic restrictions on food security, determining the vulnerable groups for food insecurity, identifying the food security resilience methods during and after COVID-19, and investigating the merits and emerging problems arising from COVID-19 prevention measures are all recommended topics for consideration in prospective studies. Considering the limitations of included studies, it is also suggested that future research should consider and evaluate the multiple food security driving factors external to COVID-19 that might increase the food security crisis.

### Registration and protocol 

This systematic review was registered in the International Prospective Register of Systematic Reviews (PROSPERO) under the registrations number: CRD42022325475. Protocol for the review process was prepared and the review was conducted as per the pre-planned protocol. The protocol is published at PLOS ONE [27] and can be accessed online (https://www.ncbi.nlm.nih.gov/pmc/articles/PMC9362949/).

### Amendments of published protocol 

The protocol of this systematic review [27] was published before the end of this article. In the protocol, the Risk of Bias in Systematic Reviews (ROBIS) tool was proposed to be used as a risk of bias assessment tool. Since this tool is more appropriate for systematic reviews than primary studies, the risk of bias of this systematic review was assessed using JBI critical appraisal checklist for cross-sectional studies.

### Supplementary Information

**Additional file 1.**

## Data Availability

All data related to this review is included in the result section of the manuscript. If any further data is needed it can be accessible via the corresponding author on request.

## References

[1] FAO (2014) Global Strategic Framework for Food Security & Nutrition (GSF). Committee on World Food Security (CFS); accessed April 2022.

[2] Ali AA, Usman AM, Badebo FB, Tilahun SH (2022) Exploring the patterns of multisectoral approach in fighting COVID-19 Pandemic in SNNPR, Ethiopia: A qualitative case study approach. PLoS One 17(2). 10.1371/journal.pone.0263667 PMC8880945 35213548

[3] Kanti MB, Satiprasad S, Poulami P, Subrata C, Abdullah A (2021). Multi-sectoral impact assessment during the 1st wave of COVID-19 pandemic in West Bengal (India) for sustainable planning and management. Arab J Geosci.

[4] Chen, Z., Cao, C. & Yang, G (2020). Coordinated multi-sectoral efforts needed to address the COVID-19 pandemic: lessons from China and the United States. Glob Health Res Policy 5, 22. 10.1186/s41256-020-00150-7 PMC7203075 32391441

[5] Syafiq A, Fikawati S, Gemily SC (2022). Household food security during the COVID-19 pandemic in urban and semi-urban areas in Indonesia. J Health Popul Nutr.

[6] Nguyen PH, Kachwaha S, Pant A, Tran LM, Ghosh S, Sharma PK, Shastri VD, Escobar-Alegria J, Avula R, Menon P (2021). Impact of COVID-19 on household food insecurity and interlinkages with child feeding practices and coping strategies in Uttar Pradesh, India: a longitudinal community-based study. BMJ Open.

[7] Alec Aaron, Anurima Baidya, Jun Wang, Christabel Chan, Erica Wetzler and Yunhee Kang (2021). The Early Impacts of COVID-19 on Food Security and Livelihood in Vietnam. Front Sustain Food Syst5. 10.1016/j.gfs.2021.100580 PMC9765004 36570721

[8] Dasgupta S, Robinson EJZ (2022). Impact of COVID-19 on food insecurity using multiple waves of high frequency household surveys. Sci Rep.

[9] Godrich SL, Lo J, Kent K, Macau F, Devine A (2022). A mixed-methods study to determine the impact of COVID-19 on food security, food access and supply in regional Australia for consumers and food supply stakeholders. Nutr J.

[10] Katoch, O. R., Sharma, R., Parihar, S., & Nawaz, A. (2023). Energy poverty and its impacts on health and education: a systematic review. Int J Energy Sector Manage.

[11] Devereux S, Béné C, Hoddinott J (2020). Conceptualising COVID-19’s impacts on household food security. Food Sec.

[12] Louie S, Shi Y, Allman-Farinelli M (2022). The effects of the COVID-19 pandemic on food security in Australia: A scoping review. Nutr Diet.

[13] Nchanji EB, Lutomia CK (2021). Regional impact of COVID-19 on the production and food security of common bean smallholder farmers in Sub-Saharan Africa: Implication for SDG's. Glob Food Sec.

[14] FAO, IFAD, UNICEF, WFP and WHO (2019) 2019 - The State of Food Security and Nutrition in the World (SOFI): Safeguarding against economic slowdowns and downturns. https://www.wfp.org/publications/2019-state-food-security-and-nutrition-world-sofi-safeguarding-against-economic; accessed on May 2022.

[15] Picchioni F, Goulao LF, Roberfroid D (2021). The impact of COVID-19 on diet quality, food security and nutrition in low- and middle-income countries: A systematic review of the evidence. Clin Nutr.

[16] Katoch OR (2022). Determinants of malnutrition among children: A systematic review. Nutrition.

[17] Kansiime MK, Tambo JA, Mugambi I, Bundi M, Kara A, Owuor C (2020). COVID-19 implications on household income and food security in Kenya and Uganda: Findings from a rapid assessment. World Dev.

[18] Niles MT, Beavers AW, Clay LA, Dougan MM, Pignotti GA, Rogus S, et al (2021). A Multi-Site Analysis of the Prevalence of Food Insecurity in the United States, before and during the COVID-19 Pandemic. Curr Dev Nutr. 5(12). 10.1093/cdn/nzab135 PMC8677520 34934898

[19] Kent K, Alston L, Murray S, Honeychurch B, Visentin D (2022). The Impact of the COVID-19 Pandemic on Rural Food Security in High Income Countries: A Systematic Literature Review. Int J Environ Res Public Health 19(6). 10.3390/ijerph19063235 PMC8954908 35328924

[20] Aborisade B, Bach C (2014). Assessing the Dimensions of Sustainable Food Security. EIJST.

[21] FAO (1996). Report of the World Food Summit. https://www.fao.org/3/w3548e/w3548e00.htm; (accessed April 2022).

[22] Rincon SJ, Dou N, Murray-Kolb LE, Hudy K, Mitchell DC, Li R, Na M (2022). Daily food insecurity is associated with diet quality, but not energy intake, in winter and during COVID-19, among low-income adults. Nutr J.

[23] Guiné RPF, Pato MLJ, Costa CAD, Costa DVTAD, Silva PBCD, Martinho VJPD (2021). Food Security and Sustainability: Discussing the Four Pillars to Encompass Other Dimensions. Foods. 10(11). 10.3390/foods10112732 PMC8622412 34829013

[24] Jafri A, Mathe N, Aglago EK, Konyole SO, Ouedraogo M, Audain K, Zongo U, Laar AK, Johnson J, Sanou D (2021). Food availability, accessibility and dietary practices during the COVID-19 pandemic: a multi-country survey. Public Health Nutr. 24(7). 10.1017/S1368980021000987 PMC8007937 33663623

[25] Pakravan-Charvadeh MR, Mohammadi-Nasrabadi F, Gholamrezai S, Vatanparast H, Flora C, Nabavi-Pelesaraei A (2021). The short-term effects of COVID-19 outbreak on dietary diversity and food security status of Iranian households (A case study in Tehran province). J Clean Prod.

[26] Page MJ, McKenzie JE, Bossuyt PM (2021). The PRISMA 2020 statement: an updated guideline for reporting systematic reviews. Syst Rev.

[27] Gebeyehu DT, East L, Wark S, Islam MS (2022) Impact of COVID-19 on the food security and identifying the compromised food security dimension: A systematic review protocol. PLoS ONE 17(8). 10.1371/journal.pone.0272859 PMC9362949 35944031

[28] Berkman ND, Lohr KN, Ansari M, McDonagh M, Balk E, Whitlock E, Reston J, Bass E, Butler M, Gartlehner G, Hartling L, Kane R, McPheeters M, Morgan L, Morton SC, Viswanathan M, Sista P, Chang S. Grading the Strength of a Body of Evidence When Assessing Health Care Interventions for the Effective Health Care Program of the Agency for Healthcare Research and Quality: An Update. Methods Guide for Comparative Effectiveness Reviews (Prepared by the RTI-UNC Evidence-based Practice Center under Contract No. 290–2007–10056-I). AHRQ Publication No. 13(14)-EHC130-EF. Rockville, MD: Agency for Healthcare Research and Quality. November 2013. https://effectivehealthcare.ahrq.gov/reports/final.cfm. (accessed May 2022)

[29] Pradeilles R, Pareja R, Creed‐Kanashiro HM, et al. (2022) Diet and food insecurity among mothers, infants, and young children in Peru before and during COVID‐19: A panel survey. Matern Child Nutr. 10.1111/mcn.13343 PMC9115223 35274825

[30] Ahmed T, Ilieva RT, Shane J, et al. (2022) A Developing Crisis in Hunger: Food Insecurity within 3 Public Colleges before and during the COVID-19 Pandemic. J Hunger Environ Nutr 1–20.

[31] Mialki K, House LA, Mathews AE (2021). Covid-19 and College Students: Food Security Status before and after the Onset of a Pandemic. Nutrients.

[32] Faridi A, Furqan M (2021). Household Characteristics, Consumption Pattern and Household Food Security Before and During Covid-19 In Banten Province. Malaysian J Public Health Med.

[33] Gaitán-Rossi P, Vilar-Compte M, Teruel G (2021). Food insecurity measurement and prevalence estimates during the COVID-19 pandemic in a repeated cross-sectional survey in Mexico. Public Health Nutr.

[34] Giacoman C, Herrera MS, Ayala Arancibia P (2021). Household food insecurity before and during the COVID-19 pandemic in Chile. Public Health.

[35] Hamadani JD, Hasan MI, Baldi AJ (2020). Immediate impact of stay-at-home orders to control COVID-19 transmission on socioeconomic conditions, food insecurity, mental health, and intimate partner violence in Bangladeshi women and their families: an interrupted time series. Lancet Glob Health.

[36] Kharroubi S, Naja F, Diab-El-Harake M (2021). Food Insecurity Pre- and Post the COVID-19 Pandemic and Economic Crisis in Lebanon: Prevalence and Projections. Nutrients.

[37] Saint Ville A, Po JYT, Sen A (2019). Food security and the Food Insecurity Experience Scale (FIES): ensuring progress by 2030. Food Secur.

[38] Sheikomar OB, Dean W, Ghattas H, et al. (2021) Validity of the Food Insecurity Experience Scale (FIES) for Use in League of Arab States (LAS) and Characteristics of Food Insecure Individuals by the Human Development Index (HDI). Curr Dev Nutr 5. 10.1093/cdn/nzab017 PMC8059488 33937614

[39] Seivwright AN, Callis Z, Flatau P (2020). Food Insecurity and Socioeconomic Disadvantage in Australia. Int J Environ Res Public Health.

[40] Aday S, Aday MS (2020). Impact of COVID-19 on the food supply chain. Food Qual Saf.

[41] Barman A, Das R, De PK (2021). Impact of COVID-19 in food supply chain: Disruptions and recovery strategy. CRBS.

[42] Harris J, Depenbusch L, Pal AA (2020). Food system disruption: initial livelihood and dietary effects of COVID-19 on vegetable producers in India. Food Secur.

[43] O’Hara JK, Woods TA, Dutton N (2021). COVID-19’s Impact on Farmers Market Sales in the Washington, D.C. Area J Agric Appl Econ.

[44] Russo RG, Ali SH, Mezzacca TA (2022). Assessing changes in the food retail environment during the COVID-19 pandemic: opportunities, challenges, and lessons learned. BMC Public Health.

[45] Béné C, Bakker D, Chavarro MJ (2021). Global assessment of the impacts of COVID-19 on food security. Glob Food Sec.

[46] Lee AJ, Patay D, Herron L-M (2021). Affordability of Heathy, Equitable and More Sustainable Diets in Low-Income Households in Brisbane before and during the COVID-19 Pandemic. Nutrients.

[47] Zhang Y, Yang K, Hou S, et al. (2021) Factors determining household-level food insecurity during COVID-19 epidemic: a case of Wuhan, China. Food Nutr Res 65. 10.29219/fnr.v65.5501 PMC7955523 33776620

[48] Yao H, Zang C, Zuo X (2022). Tradeoff analysis of the pork supply and food security under the influence of African swine fever and the COVID-19 outbreak in China. Geography and Sustainability.

[49] Pyle A, Eichinger M, Garst B, et al. (2021) Disease and disaster: Navigating food insecurity in a community affected by crises during COVID-19. JAFSCD, 1–18.

[50] Hickey GM, Unwin N (2020). Addressing the triple burden of malnutrition in the time of COVID-19 and climate change in Small Island Developing States: what role for improved local food production?. Food Secur.

[51] Tadesse G, Abate GT, Zewdie T (2020). Biases in self-reported food insecurity measurement: A list experiment approach. Food Policy.

[52] Gartlehner G, Affengruber L, Titscher V (2020). Single-reviewer abstract screening missed 13 percent of relevant studies: a crowd-based, randomized controlled trial. J Clin Epidemiol.

[53] Singh DR, Sunuwar DR, Shah SK, et al. (2021) Food insecurity during COVID-19 pandemic: A genuine concern for people from disadvantaged community and low-income families in Province 2 of Nepal. PLoS One 16, e0254954-e. 10.1371/journal.pone.0254954 PMC8294479 34288965

[54] Birner R, Blaschke N, Bosch C (2021). We would rather die from Covid-19 than from hunger- Exploring lockdown stringencies in five African countries. Glob Food Sec.

[55] Dodd W, Kipp A, Bustos M (2021). Humanitarian Food Security Interventions during the COVID-19 Pandemic in Low- and Middle-Income Countries: A Review of Actions among Non-State Actors. Nutrients.

[56] Bump JB, Friberg P, Harper DR (2021) International collaboration and covid-19: what are we doing and where are we going? BMJ 180. 10.1136/bmj.n180 PMC7842258 33509953

[57] Osendarp S, Akuoku JK, Black RE (2021). The COVID-19 crisis will exacerbate maternal and child undernutrition and child mortality in low- and middle-income countries. Nat Food.

